# Biodegradable and Renal Clearable Inorganic Nanoparticles

**DOI:** 10.1002/advs.201500223

**Published:** 2015-10-27

**Authors:** Emily B. Ehlerding, Feng Chen, Weibo Cai

**Affiliations:** ^1^ Department of Medical PhysicsUniversity of WisconsinMadisonWI53706USA; ^2^ Department of RadiologyUniversity of WisconsinMadisonWI53706USA; ^3^ University of Wisconsin Carbone Cancer CenterMadisonWI53706USA

**Keywords:** biodegradable, inorganic nanoparticles, molecular imaging, renal clearance, theranostics

## Abstract

Personalized treatment plans for cancer therapy have been at the forefront of oncology research for many years. With the advent of many novel nanoplatforms, this goal is closer to realization today than ever before. Inorganic nanoparticles hold immense potential in the field of nano‐oncology, but have considerable toxicity concerns that have limited their translation to date. In this review, an overview of emerging biologically safe inorganic nanoplatforms is provided, along with considerations of the challenges that need to be overcome for cancer theranostics with inorganic nanoparticles to become a reality. The clinical and preclinical studies of both biodegradable and renal clearable inorganic nanoparticles are discussed, along with their implications.

## 1 Introduction

With one in four deaths in the United States being attributed to cancer, the diagnosis and treatment of cancer is one of the main focuses of the biomedical world. Great progress has been made in the last few decades, with overall death rates declining by 20% since 1991.^1^ However, with over 500 000 deaths due to cancer in 2014 alone, there is still an enormous need for the development of better cancer treatments today. Personalized medicine has recently come to the forefront of cancer therapy discussions, representing a paradigm shift from generalized treatments to ones that are developed specifically for an individual's cancer thumbprint. By targeting the molecular characteristics of each unique cancer, a treatment regimen can ideally be developed to allow for a maximized therapeutic index. However, challenges exist in the development of these biomarker‐targeted agents.^2^

The field of nanotechnology holds great promise in cancer imaging and therapy, as novel nanoplatforms for biomedical applications are being developed at an impressive rate.^3^ Specifically, several inorganic platforms have been created that demonstrate potential in both preclinical and clinical trials.^4^, ^5^, ^6^, ^7^, ^8^, ^9^, ^10^, ^11^ Due to fast and high uptake in the reticuloendothelial system (RES; e.g. liver and spleen), nanoparticles with large particle size (>10 nm) or heavy metal components have provoked increased long‐term toxicity concerns.^12^ Thus, most nanoparticles that have found their way into human clinical trials have been limited to those which are organic‐ or polymeric‐based.^13^ Liposomal constructs of many chemotherapeutics have been approved by the US Food and Drug Administration (FDA).^14^, ^15^, ^16^ Abraxane, a novel 130‐nm, albumin‐bound particle form of paclitaxel, has also been designed to utilize endogenous albumin pathways to increase intratumor concentrations of the active drug.^17^ Promising multifunctional agents known as porphysomes (formed by the super‐assembly of porphyrin‐phospholipids) have recently been demonstrated as photothermal, photodynamic, and photoacoustic enhancement agents, as well as drug delivery vehicles.^18^, ^19^, ^20^ Ferritin, a ubiquitous protein in most living beings, has been utilized as a biodegradable platform for multimodality imaging.^21^ While these and many other organic nanoplatforms are being explored preclinically, immense obstacles still stand in the way of further clinical translation, including monetary and time considerations.

Recent preclinical research has shown the usefulness of inorganic platforms in cancer theranostics. However, only those with biodegradable or renal clearable properties have reasonable chances for potential clinical translation. In this review article, the clinical and preclinical studies of both biodegradable and renal clearable inorganic nanoparticles are discussed, along with the concerns (or limitations) that hold them back from human trials and how these are being addressed.

## 2 Toxicity Concerns of Inorganic Nanoplatforms

Inorganic nanoplatforms face larger challenges in the process to clinical translation when compared to organic systems. Most of these nanoparticles are made from materials that are well‐characterized in their bulk state. However, the nanoscale of these structures creates additional properties that must be considered when analyzing their toxicity.^12^, ^22^ The same properties that give nanoplatforms their promise in medicine may also be the most crucial to consider in toxicological evaluations.

For example, traditional cytotoxicity assays may not be proper techniques for nanoparticles, as the particles themselves have been found to chemically reduce the dyes (e.g. MTT (3‐[4,5‐dimethylthiazol‐2‐yl]‐2,5‐diphenyltetrazolium bromide)) used in classical assays, resulting in inaccurate toxicity determinations.^23^ The majority of existing toxicological data results from in vitro studies, but will need to be validated with in vivo experimentation for a reasonably long time period.^24^ Most importantly, a standardized method of toxicity determination for nanoplatforms is necessary to facilitate further clinical translation. Indeed, the scientific community as a whole has recognized the need for nanoplatform safety standards, initiating the implementation of databases for techniques of nanotechnology characterization and measurement.^25^

**Scheme 1:**
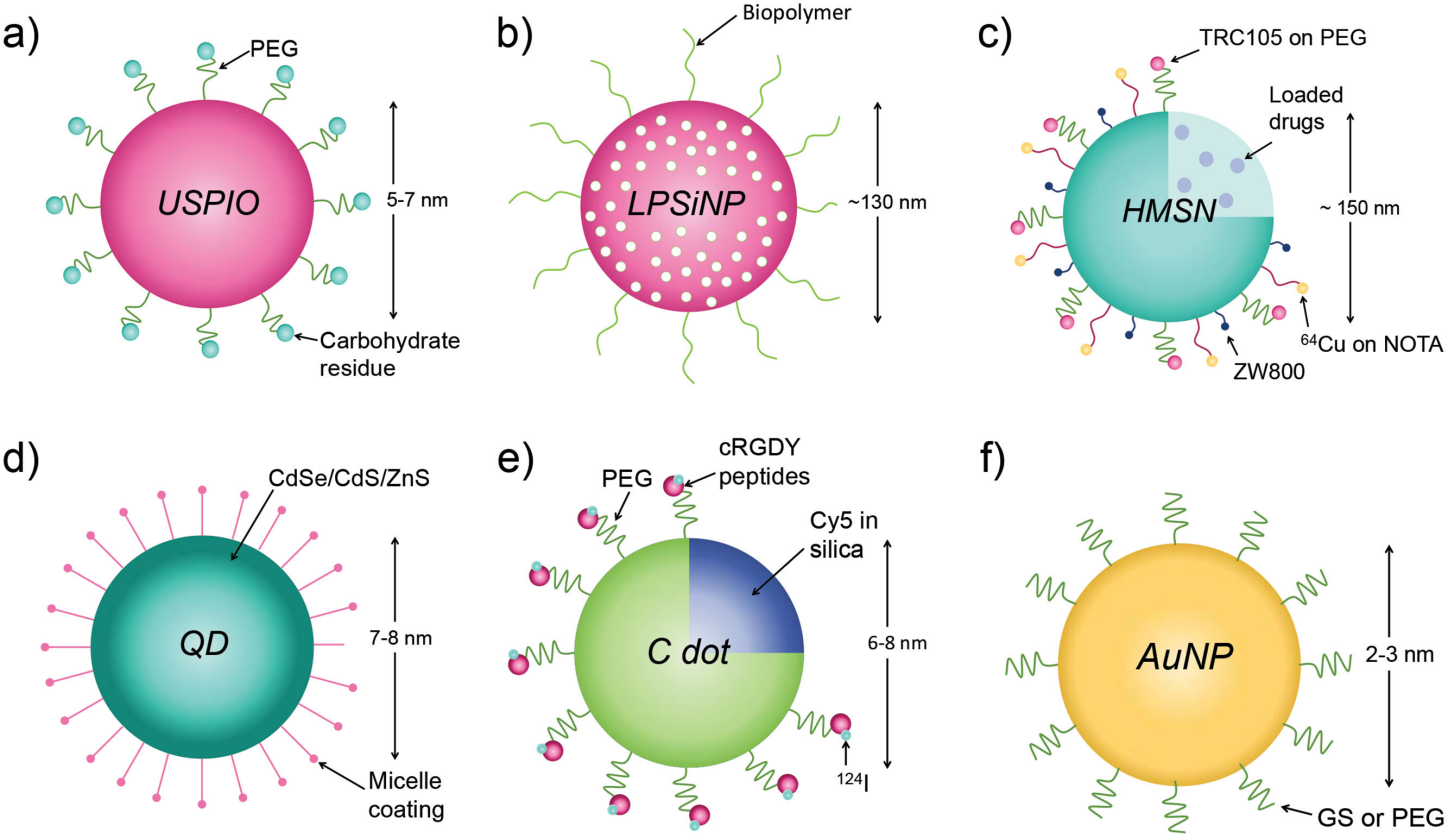
Schematic illustrations of biodegradable (a–c) and renal clearable inorganic nanoparticles (d–f). a) Ultra‐small superparamagnetic iron oxide (USPIO) nanoparticle for magnetic resonance imaging. b) Biodegradable biopolymer coated luminescent porous silicon nanoparticles (LPSiNP). c) Functionalized hollow mesoporous silica nanoparticle (HMSN) for positron emission tomography imaging, optical imaging, and tumor targeted drug delivery. d) Renal clearable cysteine coated quantum dot (QD). e) Ultra‐small fluorescent silica nanoparticle (also known as C dot) functionalized with targeting ligands (cRGDY), poly(ethylene glycol) and radioisotope (^124^I) for tumor targeted multimodality imaging. f) Glutathione (GS), or poly(ethylene glycol) (PEG) coated 2–3 nm sized AuNPs.

One recent study evaluated the toxicity of phospholipid micelle‐encapsulated CdSe/CdS/ZnS quantum dots (QDs) in rhesus macaques.^8^ Results showed that after ninety days blood and biochemical markers were within normal ranges, indicating that the immediate effects of as‐studied QDs are not the primary concern—the longer‐lasting impacts are crucial. The majority of the QDs were found to be retained in the RES even after the ninety‐day trial. These long‐term effects of nanoparticles within the body are perhaps the most fundamental areas to be studied. Since the FDA has declared that all injected imaging agents must be cleared in a reasonable amount of time, engineering of nanomaterials to allow for biodegradation or renal clearance is crucial to avoid long‐term retention.^10^ However, this goal is counteracted by the fact that nanoparticles are often inherently stable, even maintaining their fluorescent abilities after two years in vivo in a QD‐related study.^26^ Many techniques and novel nanoplatforms have been explored in the last few years for biologically safe theranostic nanomedicine, resulting in two categories of promising inorganic nanoplatforms: biodegradable larger sized nanoparticles and renal clearable ultra‐small sized nanoparticles (**Scheme** **1**).

## 3 Biodegradable Nanoparticles

### 3.1 Iron Oxide Nanoparticles

Iron oxide nanoparticles (IONPs) hold great promise in widespread medical applications as magnetic resonance imaging (MRI) contrast agents and in drug delivery, hyperthermia, and immunoassays.^27^ When compared to QDs which contain heavy metals, iron‐based nanoparticles are known to be less toxic given that iron ions (the biodegradation byproduct of IONPs) are essential as a trace element and the iron transport pathways and regulation of iron homeostasis are quite well understood.^28^ Preclinical studies indicated that iron levels in rats returned to normal values within three weeks or more after intravenous administration of oleic acid–pluronic‐coated iron oxide nanoparticles.^29^ Research further showed that cirrhosis and hepatocellular carcinoma were not evident until the liver iron concentration reached over ten times the natural level, which was far beyond any required dose.^30^ Since the human body naturally contains a substantial level of iron, the small concentration of free iron that results from the degradation of iron oxide nanoparticles is not thought to have biologically detrimental impacts.

Ferumoxides (Endorem in Europe, Feridex in the USA, particle size: 120–180 nm) and ferucarbotran (Resovist, particle size: ≈60 nm) are two negative magnetic resonance contrast agents based on iron oxide nanoparticles that were previously approved for clinical use.^31^, ^32^ Most current clinical trials related to IONPs are focusing on the anemia drug ferumoxytol (Feraheme, AMAG Pharmaceuticals), which is an iron replacement product containing non‐stoichiometric magnetite coated with polyglucose sorbitol carboxymethylether (particle size range 17–31 nm).^33^ In March 2015, the FDA ordered stricter warnings and contraindications for Feraheme, stating that potentially fatal allergic reactions can occur with this drug in patients who have had an allergic reaction to any intravenous iron replacement product.^34^

Besides larger sized iron oxide nanoparticles, ultra‐small superparamagnetic iron oxide nanoparticles have also been developed as an alternative magnetic resonance contrast agent for patients at risk for nephrogenic systemic fibrosis.^35^ By combing high‐resolution MRI with 2–3 nm sized lymphotropic IONPs, successful detection of small and otherwise undetectable lymph‐node metastases in patients with prostate cancer has also been achieved.^36^ Although iron oxide nanoparticles with different sizes have been approved for use in the past (**Table** **1**), they are no longer available (due to the toxicity concerns) with the exception of the oral iron oxide contrast agent, Lumirem/Gastromark (approved by the FDA in 1996).

**Table 1:** Selected inorganic nanoparticles in clinical and preclinical studies

Nanoparticle Description (Trade Name)	Application	Development Status	References
Carboxydextran‐coated iron oxide nanoparticles (Resovist)	MRI contrast agent	Production was abandoned in 2009	32
Ultra‐small superparamagnetic iron oxide nanoparticles (Clariscan)	MRI contrast agent	Discontinued due to safety concerns	59
Aminosilane‐coated iron oxide nanoparticles (NanoTherm)	Thermal therapy	Phase 1	60
Luminescent porous silicon nanoparticles	Imaging and drug delivery	Preclinical studies	9
Mesoporous silica nanoparticles	Imaging and drug delivery	Preclinical studies	7, 41, 42, 43
Ultra‐small quantum dots	Imaging	Preclinical studies	53, 54, 61
Ultra‐small gold nanoparticles	Imaging	Preclinical studies	6, 48, 50
Ultra‐small fluorescent silica nanoparticles	Image‐guided surgery	Investigational New Drug approved (FDA)	4, 5

### 3.2 Porous Silicon and Silica Nanoparticles

**Figure 1:**
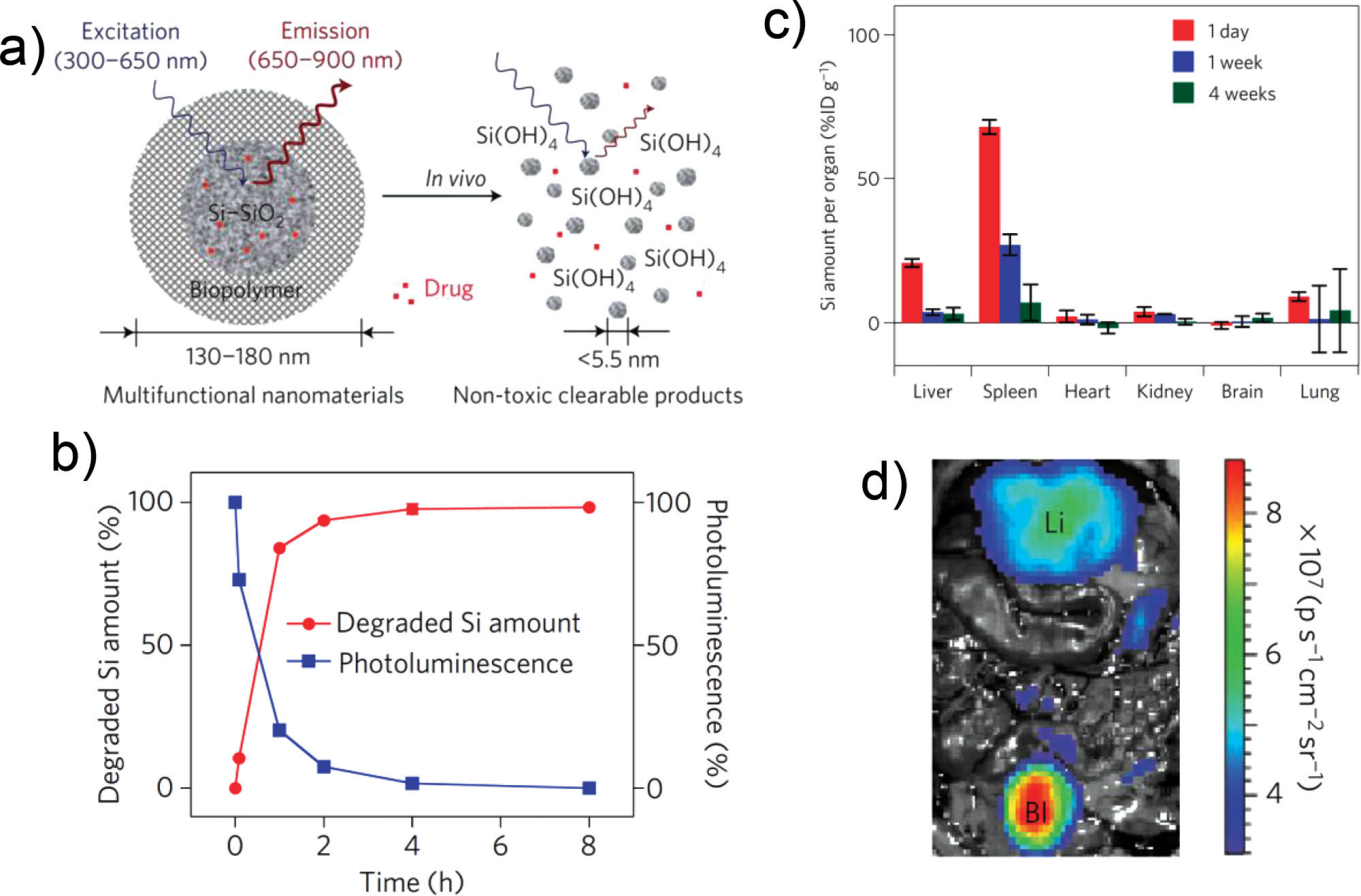
a) Schematic illustration of in vivo degradation process of the biopolymer‐coated LPSiNPs. b) Degradation and change of photoluminescence when incubating LPSiNPs in PBS at 37 °C. c) In vivo biodistribution and biodegradation of LPSiNPs over a period of 4 weeks. d) In vivo image showing the clearance of LPSiNPs into the bladder at 1 h post injection. Li (liver) and Bl (bladder). Reproduced with permission.^9^ Copyright 2009, Nature Publishing Group.

Like iron, silicon is a trace element within the human body. Porous silicon nanoparticles have been formulated to be biodegraded into renal clearable components while effectively evading the RES (**Figure** **1**).^9^ The intact particles themselves are on the scale of 130–180 nm, which would traditionally be eliminated through the RES. However, by degrading into water‐soluble silicic acid (i.e. Si(OH)_4_) (Figure 1a,b), the injected luminescent porous silicon nanoparticles (LPSiNPs) were found to clear noticeably within a week (Figure 1c). In vivo near‐infrared (NIR) imaging further confirmed the clearance of LPSiNPs into the bladder at 1 h post injection (Figure 1d). As developed LPSiNPs have been further demonstrated to effectively carry drug payloads, provide NIR luminescence, and self‐destruct into the subparts that demonstrate very minor toxicity. Although solid evidence has been provided to support the degradation of LPSiNPs while circulating in the blood stream, a reliable imaging technique that can be used for visualizing the degradation process is currently unavailable. Engineering of LPSiNPs with tunable and controllable in vivo biodegradation rate is vital to prevent the self‐destruction of LPSiNPs before delivering the drug payloads to the target of interest.

**Figure 2:**
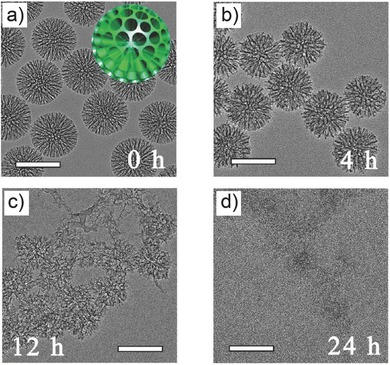
TEM images showing the degradation of MSN (pore size: ≈10 nm) in simulated body fluid (SBF) at 37 °C. a) 0 h, b) 4 h, c) 12 h and d) 24 h after soaking in SBF. Scale bar: 100 nm. Reproduced with permission.^40^ Copyright 2014, American Chemical Society.

Silica (or silicon dioxide) is “generally recognized as safe” by the FDA (ID Code: 14808–60–7),^37^ which is highly desirable for future clinical translation.^4^, ^5^ Biocompatible mesoporous silica nanoparticles (MSNs) have been accepted as one of the most attractive inorganic drug delivery systems due to their high specific surface area and pore volume.^38^, ^39^ Very recently, a dendritic mesoporous silica structure with tunable pore size (ranging from 2–13 nm) was developed, and showed a faster biodegradation rate (byproduct: silicic acid) in simulated body fluid for MSN with a larger pore size (≈10 nm, **Figure** **2**).^40^ In another study, mesoporous organosilica nanoparticles (MONs) with a large pore size (8–13 nm) were developed by using a micelle/precursor co‐templating assembly strategy.^41^ Hollow MONs with up to five different organic hybridizations were further created for high intensity focused ultrasound‐responsive drug‐release by the same research group.^42^ As‐developed HMONs maintained significant biocompatibility and demonstrated tunable biodegradation rates. Although radiolabeled‐ and antibody‐conjugated MSN (or hollow MSN) with a smaller pore size range (2–3 nm) have been developed for in vivo tumor vasculature targeted imaging and drug delivery,^7^, ^43^ engineering of biodegradable MSN (with a larger pore size and controllable degradation rate) for in vivo tumor targeted imaging and therapy has not been achieved yet.

Biodegradation into nontoxic subparts is an ideal property for injected inorganic nanoparticle based drug delivery systems, as long‐term toxicity of the carriers would be of less concern. However, the carriers also need to be stable enough in vivo to allow for sufficient tumor targeting to be achieved. Remote stimuli (such as magnetic field, light, heat or ultrasound) responsive drug delivery is another approach for reducing the potential toxicity, and has attracted increasing interest in the research community.^44^ Although organic‐based or hybrid nanoplatforms, such as dendron–virus complexes^45^ and magnetoferritin,^46^ have been developed for controlled assembly (or disassembly), delivery and release of compounds in vivo, the engineering of inorganic nanoplatforms that can be stimulated disassembled is still a huge challenge due to their rigid and crystallized nanostructure.

## 4 Renal Clearable Nanoparticles

### 4.1 Quantum Dots

Ultra‐small inorganic nanoparticles (less than 10 nm) together with suitable surface modifications can naturally be cleared through the renal system after administration.^47^ Poly(ethylene glycol) (PEG) and ziwitterionic ligand coating are two of the widely used surface modification techniques for creating renal clearable nanoparticles with a neutral surface charge.^10^, ^48^ Since the pioneer work of renal clearable QDs in 2007,^10^ other inorganic nanoparticles such as ultra‐small fluorescent silica nanoparticles (C dots),^4^, ^5^, ^49^ glutathione‐coated gold nanoparticles,^6^, ^50^ and carbon nanotubes^51^ have also been developed. So far, imaging or image‐guided surgery are the major applications for these renal clearable inorganic nanoparticles.

**Figure 3:**
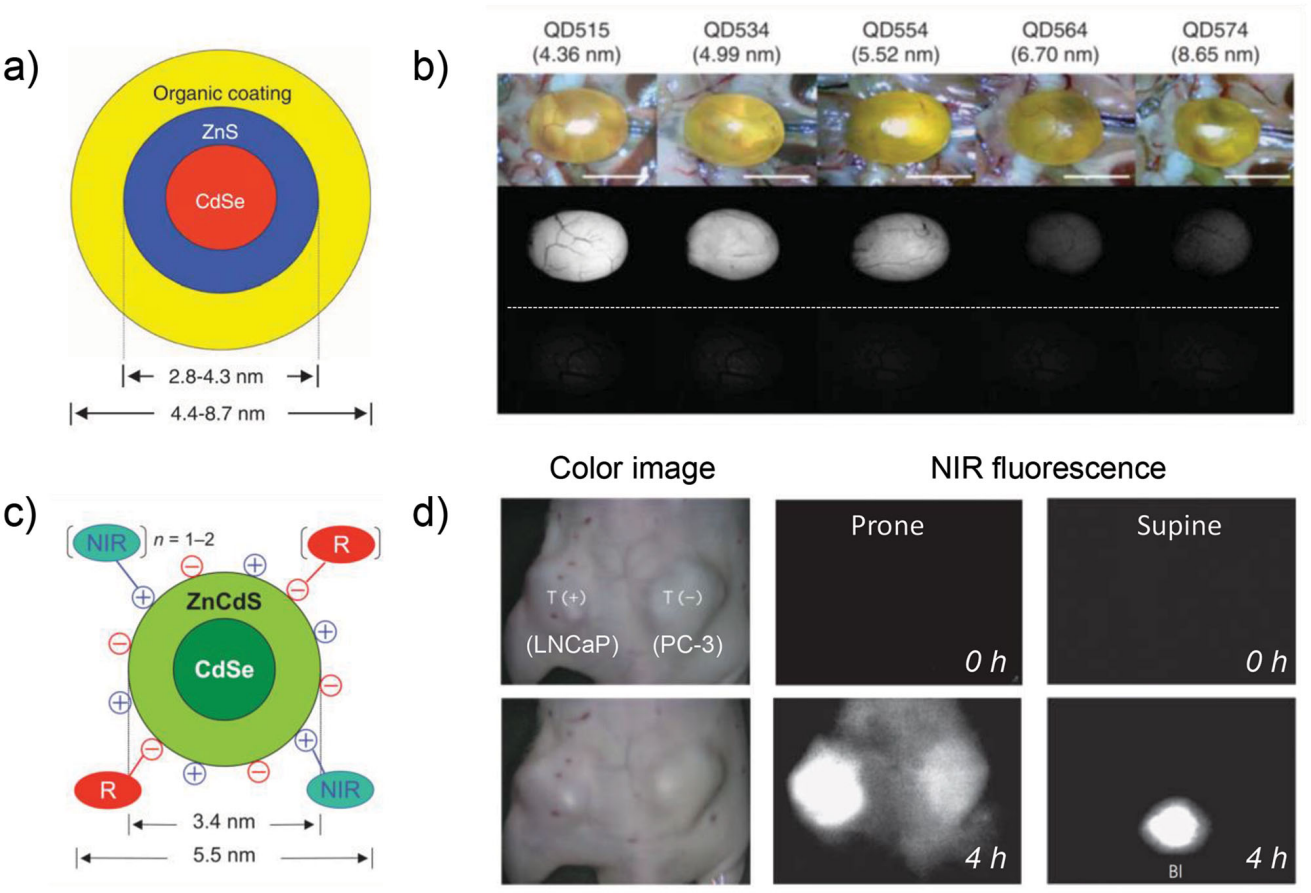
a) Schematic illustration of ultra‐small QDs. b) Renal clearable cutoff study. Top: color photos of un‐injected bladders. Bottom: fluorescence images of un‐injected bladders. Middle: fluorescence images at 4 h post injection of different QDs (hydrodynamic size range: 4–8 nm). Reproduced with permission.^10^ Copyright 2007, Nature Publishing Group. c) A schematic illustration of ultra‐small QDs functionalized with targeting ligands and NIR) fluorophores. d) In vivo NIR fluorescence imaging of GPI (a ligand which targets to prostate‐specific membrane antigen [PSMA]) and 800‐CW functionalized QDs (or QD‐CW‐GPI) in PSMA positive (LNCaP) and negative (PC‐3) tumor models. Reproduced with permission.^11^ Copyright 2010, Nature Publishing Group.

QDs have found their way into preclinical biomedical applications as fluorescent labels.^52^ Their unique size‐tunable light emission/absorption properties and high quantum yield make QDs one of the best inorganic nanoparticles for optical imaging, if they can be effectively concentrated within the region of interest. QDs with a final hydrodynamic (HD) size of 5.5 nm were developed that allowed for efficient renal clearance in preclinical studies (**Figure** **3**a,b).^10^ Many approaches have been taken to generate QDs that are both actively and passively targeted to specific biological targets. For example, maleimide‐activated QDs can be conjugated to the thiols of reduced antibodies and ligands to generate targeted nanoparticles.^53^ Due to the fast clearance rate (within hours), careful design considerations (particle size, surface charge, number of targeting ligands, etc.) need to be taken into account to generate renal clearable QDs that can still actively target to specific biological targets in vivo (Figure 3c, d).^11^ Recent advances in developing self‐illuminating QDs by conjugating with *Renilla reniformis* 54 also make it possible to create renal clearable and self‐illuminating QDs for future deep‐tissue, multiplexed, in vivo imaging. Since QDs are traditionally made of heavy metals (such as cadmium), toxicity concerns have greatly limited their clinical translation, and so far no first‐in‐human clinical trials have been approved by the FDA.

## 4.2 C dots

**Figure 4:**
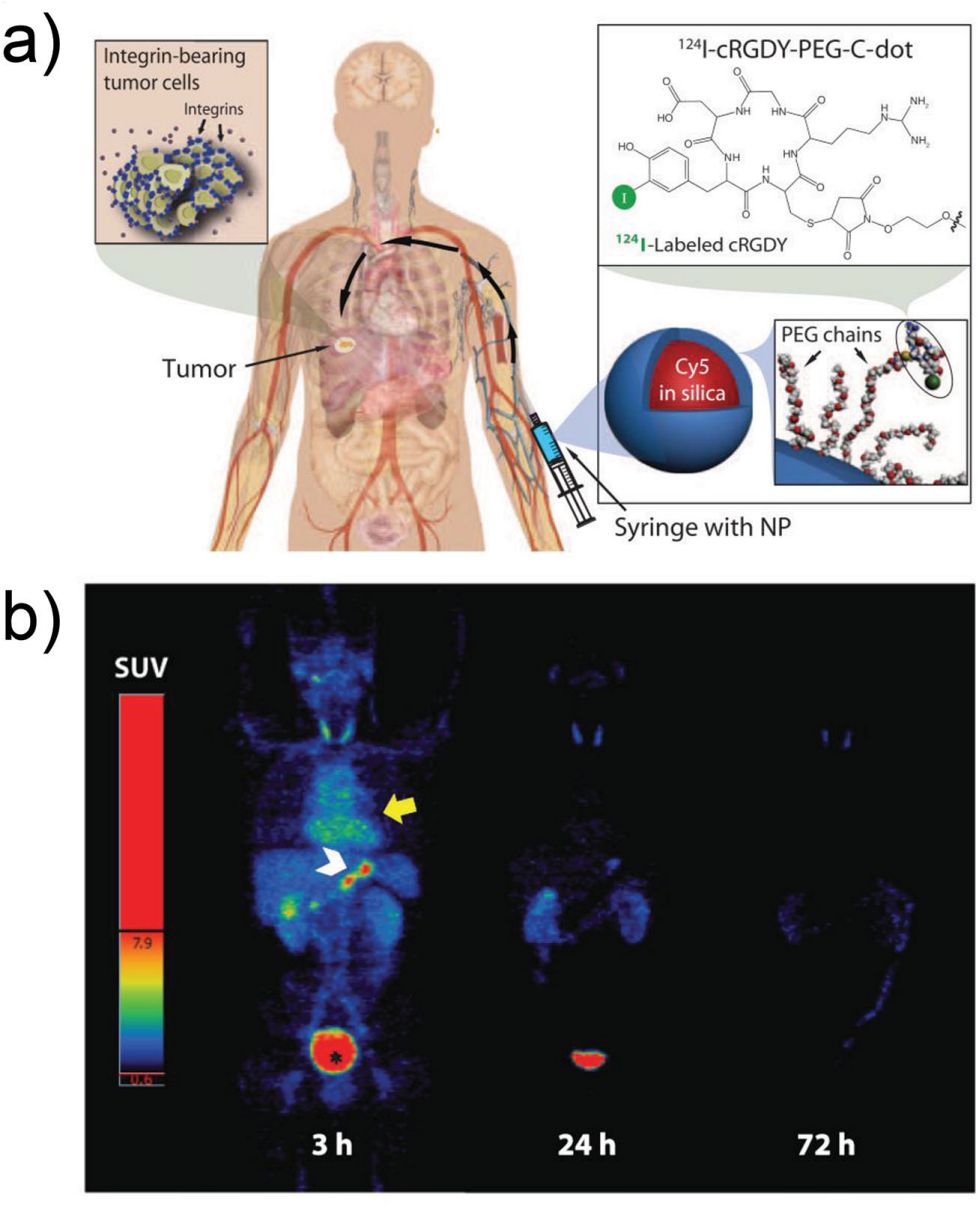
a) Schematic illustration of the use of cRGDY peptide functionalized, ^124^ I‐labeled C dot as hybrid (PET‐optical) imaging nanoparticle (^124^I‐cRGDY–PEG–C dots) in a human patient. b) Maximum intensity projection PET images at 3, 24, and 72 h after intravenous injection of ^124^I‐cRGDY–PEG–C dots revealed probe activity in bladder (*), heart (yellow arrow), and bowel (white arrowhead). Reproduced with permission.^5^ Copyright 2014, American Association for the Advancement of Science.

Ultra‐small dye encapsulated fluorescence silica nanoparticles, known as C dots (or “Cornell dots”), found their way into clinical trials in January 2011 (NCT01266096, NCT02106598).^4^, ^5^ These ultra‐small dense silica nanostructures are modified with near NIR dyes, cRGD, and radioisotopes, allowing for multimodality targeted imaging of cancer (**Figure** **4**a).^4^ With HD sizes of 6–8 nm, C dots have sufficiently long in vivo blood circulation time (t_1/2_ = 190 min) to allow for optical imaging to be feasible throughout an entire surgery procedure.^49^ PEG surface modification changes the highly negative charged surface to be neutral, which prevents C dots from forming large aggregates when circulating in the blood, and allows them to be efficiently cleared through the renal system.^49^ The NIR emission of the encapsulated dyes allows for minimized signal attenuation and autofluorescence from surrounding tissue, resulting in improved signal‐to‐noise ratio and a maximal contrast per administered dose. Iodine‐124 (t_1/2_ = 4.18 d) was also conjugated to the surface of the C dots for positron emission tomography (PET) verification of the optical images (Figure 4b). Initial results of clinical trials in patients with metastatic melanoma indicate no adverse effects due to the nanoparticles.^4^ A more detailed follow‐up clinical trial focused on the safety, pharmacokinetics, clearance properties, and radiation dosimetry of PET‐optical hybrid ^124^I‐cRGDY–PEG–C dots (Figure 4b).^5^ Preliminary clinical trials showed that C dots could not only significantly improve target‐to‐background ratio (when compared with free dyes), but also showed higher sensitivity and specificity (when compared with ^18^F‐fluorodeoxyglucose [^18^F‐FDG]),^5^ indicating great potential for safe use of these particles in human cancer diagnostics in the near future.

### 4.3 Ultra‐Small Gold Nanoparticles

**Figure 5:**
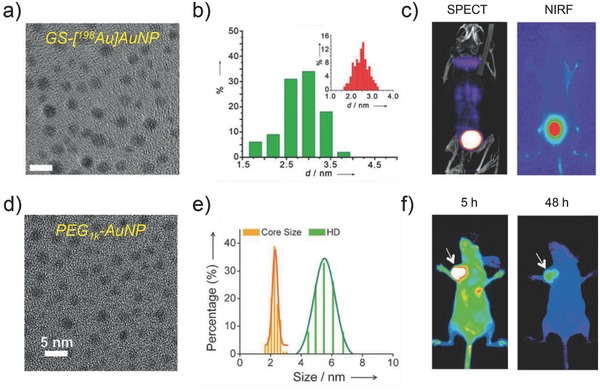
a) TEM image of GS‐[^198^Au]AuNP. Scale bare: 5 nm. b)ynamic light scattering analysis of GS‐[^198^Au]AuNP showing a HD size of 3.0 ± 0.4 nm. Core size: 2.6 ± 0.3 nm (inset). c) In vivo SPECT (left) and fluorescence images (right) of balb/c mice after tail vein injection of GS‐[^198^Au]AuNP 1 h post injection. Reproduced with permission.^50^ d) TEM image of PEG_1k_‐AuNP. e) Dynamic light scattering analysis of PEG_1k_‐AuNP showing a HD size of 5.5 ± 0.4 nm. Core size: 2.3 ± 0.3 nm (inset). f) In vivo NIR fluorescence imaging of PEG_1k_‐AuNP in MCF‐7 tumor‐bearing nude mice after tail veil injection at 5 and 48 h (EPR based passive targeting). Reproduced with permission.^48^

Fluorescent ultra‐small gold nanoparticles (AuNPs) with efficient renal clearance capability have also been developed recently.^6^, ^48^, ^55^, ^56^ Glutathione coated 2–3 nm sized AuNPs were chelator‐free labeled with gold‐198 (^198^Au) to form Glutathione (GS)‐[^198^Au]AuNPs (**Figure** **5**a,b). Over 50% of the particles were found to be excreted in the urine within 24 h, by tracking with single‐photon emission computed tomography (SPECT) and optical imaging (Figure 5c).^50^ In a follow‐up study, PEGylated AuNPs (PEG molecular weight: 1 kDa) were demonstrated to have three times higher passive targeting efficacy when compared with GS‐AuNPs (Figure 5d–f).^48^ Although tumor necrosis factor bound colloidal gold nanoparticles have been approved for a phase I clinical trial (NCT00356980, NCT00436410),^57^ and silica‐Au nanoshell (also known as Auroshell) also gained approval in a clinical pilot thermal treatment for patients with refractory and/or recurrent tumors of the head and neck (NCT00848042, NCT01679470), the clinical translation of ultra‐small renal clearable AuNPs is still in the early stages.

## 5 Challenges and Future Directions

Individualized treatment has become a goal of oncology research in recent years. Inorganic nanoparticles with biodegradable and renal clearable properties could have an optimistic outlook in future nano‐oncology by offering safer and multifunctional nanosystems for both cancer diagnosis and therapy. Table 1 further provides a collection of representative inorganic nanoparticles that have been used in clinical and preclinical studies.

Toxicity concerns will continue to be one of the largest obstacles during the clinical translation of inorganic nanoparticles. A great deal of preclinical work has yet to be done in determining the best method for characterizing and reducing toxic effects. For inorganic nanoparticles that are mainly composed of metals that are foreign to the human body, renal clearance has been demonstrated to be the most practical means for evading undesirable toxic effects. Sophisticated control of particle size and surface coating is vital, since these characteristics determine the pharmacokinetics and biodegradation properties of these nanoparticles. Although a number of renally‐clearable particles have been developed to date,^6^, ^10^, ^11^, ^48^, ^50^ C dots are the only renal clearable inorganic nanoparticles that have gained Investigational New Drug approval by the FDA for first‐in‐human clinical trials.^4^, ^5^

One of the major challenges facing renal clearable inorganic nanoparticles is the specific accumulation in tumor tissues in vivo. The fast clearance in vivo through the renal system indeed is beneficial for reducing the toxicity concerns, but in vivo active targeting to tumor tissue needs to be further explored for their application to be viable.^11^, ^55^ We believe the use of these renal‐clearable nanoparticles has the potential to revolutionize future cancer detection, image‐guided surgery and treatment provided that a satisfied balance between in vivo targeting and renal clearance can be achieved. Biodegradable porous silicon (or silica) based nanoparticles may hold greater potential for future clinical translation when compared with other inorganic drug delivery systems considering the successful clinical translation of C dots,^4^, ^5^ the well‐established surface modification chemistry,^58^ the demonstrated tumor targeted drug delivery capabilities,^7^, ^43^ and their potential in alleviating the long‐term RES accumulation concerns.

## 6 Conclusions

In conclusion, the clinical and preclinical studies of biodegradable and renal clearable inorganic nanoparticles are explored in this short review article. Although toxicity concerns still remain as the major barriers to further translation of inorganic nanoparticles from bench to the bedside, progress has been made in demonstrating the reduced toxicity effects by engineering biodegradable and renal clearable nanoparticles while maintaining their desirable imaging or therapeutic functionalities. Personalized treatments are not so much a dream as a real possibility with the great advances that have been made in the field of nano‐oncology. We believe inorganic nanoparticles with biodegradable and renal clearable properties could hold great potential to revolutionize future cancer diagnosis and therapy.

## References

[1] R. Siegel , J. Ma , Z. Zou , A. Jemal , Ca‐Cancer J. Clin. 2014, 64, 9. 24399786 10.3322/caac.21208

[2] I. I. Wistuba , J. G. Gelovani , J. J. Jacoby , S. E. Davis , R. S. Herbst , Nat. Rev. Clin. Oncol. 2011, 8, 135. 21364686 10.1038/nrclinonc.2011.2

[3] A. S. Thakor , S. S. Gambhir , Ca‐Cancer J. Clin. 2013, 63, 395. 24114523 10.3322/caac.21199

[4] M. Benezra , O. Penate‐Medina , P. B. Zanzonico , D. Schaer , H. Ow , A. Burns , E. DeStanchina , V. Longo , E. Herz , S. Iyer , J. Wolchok , S. M. Larson , U. Wiesner , M. S. Bradbury , J. Clin. Invest. 2011, 121, 2768. 21670497 10.1172/JCI45600 PMC3223837

[5] E. Phillips , O. Penate‐Medina , P. B. Zanzonico , R. D. Carvajal , P. Mohan , Y. Ye , J. Humm , M. Gönen , H. Kalaigian , H. Schöder , H. W. Strauss , S. M. Larson , U. Wiesner , M. S. Bradbury , Sci.Transl. Med. 2014, 6, 260ra149. 10.1126/scitranslmed.3009524 PMC4426391 25355699

[6] C. Zhou , M. Long , Y. Qin , X. Sun , J. Zheng , Angew. Chem. Int. Ed. 2011, 50, 3168. 10.1002/anie.201007321 PMC3095852 21374769

[7] F. Chen , H. Hong , S. Shi , S. Goel , H. F. Valdovinos , R. Hernandez , C. P. Theuer , T. E. Barnhart , W. Cai , Sci. Rep. 2014, 4, 5080. 24875656 10.1038/srep05080 PMC4038837

[8] L. Ye , K.‐T. Yong , L. Liu , I. Roy , R. Hu , J. Zhu , H. Cai , W.‐C. Law , J. Liu , K. Wang , J. Liu , Y. Liu , Y. Hu , X. Zhang , M. T. Swihart , P. N. Prasad , Nat. Nano. 2012, 7, 453. 10.1038/nnano.2012.74 22609691

[9] J.‐H. Park , L. Gu , G. von Maltzahn , E. Ruoslahti , S. N. Bhatia , M. J. Sailor , Nat. Mater. 2009, 8, 331. 19234444 10.1038/nmat2398 PMC3058936

[10] H. Soo Choi , W. Liu , P. Misra , E. Tanaka , J. P. Zimmer , B. Itty Ipe , M. G. Bawendi , J. V. Frangioni , Nat. Biotech. 2007, 25, 1165. 10.1038/nbt1340 PMC2702539 17891134

[11] H. S. Choi , W. Liu , F. Liu , K. Nasr , P. Misra , M. G. Bawendi , J. V. Frangioni , Nat. Nanotechnol. 2010, 5, 42. 19893516 10.1038/nnano.2009.314 PMC2797834

[12] A. Nel , T. Xia , L. Mädler , N. Li , Science 2006, 311, 622. 16456071 10.1126/science.1114397

[13] A. Z. Wang , R. Langer , O. C. Farokhzad , Annu. Rev. Med. 2012, 63, 185. 21888516 10.1146/annurev-med-040210-162544

[14] J. Lao , J. Madani , T. Puertolas , M. Alvarez , A. Hernandez , R. Pazo‐Cid , A. Artal , A. A. Torres , J. Drug Delivery 2013, 2013, 456409. 10.1155/2013/456409 PMC3619536 23634302

[15] J. Silverman , S. Deitcher , Cancer Chemother. Pharmacol. 2013, 71, 555. 23212117 10.1007/s00280-012-2042-4 PMC3579462

[16] Y. Barenholz , J. Controlled Release 2012, 160, 117. 10.1016/j.jconrel.2012.03.020 22484195

[17] M. R. Green , G. M. Manikhas , S. Orlov , B. Afanasyev , A. M. Makhson , P. Bhar , M. J. Hawkins , Ann. Oncol. 2006, 17, 1263. 16740598 10.1093/annonc/mdl104

[18] J. F. Lovell , C. S. Jin , E. Huynh , H. Jin , C. Kim , J. L. Rubinstein , W. C. W. Chan , W. Cao , L. V. Wang , G. Zheng , Nat. Mater. 2011, 10, 324. 21423187 10.1038/nmat2986

[19] C. S. Jin , J. F. Lovell , J. Chen , G. Zheng , ACS Nano 2013, 7, 2541. 23394589 10.1021/nn3058642 PMC3610399

[20] J. F. Lovell , T. W. Liu , J. Chen , G. Zheng , Chem. Rev. 2010, 110, 2839. 20104890 10.1021/cr900236h

[21] X. Lin , J. Xie , L. Zhu , S. Lee , G. Niu , Y. Ma , K. Kim , X. Chen , Angew. Chem. Int. Ed. 2011, 50, 1569. 10.1002/anie.201006757 PMC3629976 21308906

[22] B. J. Marquis , S. A. Love , K. L. Braun , C. L. Haynes , Analyst 2009, 134, 425. 19238274 10.1039/b818082b

[23] T. Laaksonen , H. Santos , H. Vihola , J. Salonen , J. Riikonen , T. Heikkilä , L. Peltonen , N. Kumar , D. Y. Murzin , V.‐P. Lehto , J. Hirvonen , Chem Res Toxicol 2007, 20, 1913. 17990852 10.1021/tx700326b

[24] H. C. Fischer , W. C. W. Chan , Curr. Opin. Biotechnol. 2007, 18, 565. 18160274 10.1016/j.copbio.2007.11.008

[25] ISO, in ISO, IEC, NIST, OECD Int. Workshop Documentary Standards Measurement Characterization Nanotechnol., Gaithersburg, MD, USA, 2008.

[26] B. Ballou , L. A. Ernst , S. Andreko , T. Harper , J. A. J. Fitzpatrick , A. S. Waggoner , M. P. Bruchez , Bioconjugate Chem. 2007, 18, 389. 10.1021/bc060261j 17263568

[27] A. K. Gupta , M. Gupta , Biomaterials 2005, 26, 3995. 15626447 10.1016/j.biomaterials.2004.10.012

[28] P. C. Wang , L. Shan , J. Basic Clin. Med. 2012, 1, 1. 24159426 PMC3805053

[29] T. K. Jain , M. K. Reddy , M. A. Morales , D. L. Leslie‐Pelecky , V. Labhasetwar , Mol. Pharmaceutics 2008, 5, 316. 10.1021/mp7001285 18217714

[30] M. L. Bassett , J. W. Halliday , L. W. Powell , Hepatology 1986, 6, 24. 3943787 10.1002/hep.1840060106

[31] K. Leung , in Molecular Imaging and Contrast Agent Database (MICAD), National Center for Biotechnology Information (US), MD, USA, 2004–2013. 20641179

[32] P. Reimer , T. Balzer , Eur. J. Radiol 2003, 13, 1266. 10.1007/s00330-002-1721-7 12764641

[33] ClinicalTrials.gov: a web‐based resource that provides patients, their family members, health care professionals, researchers, and the public with easy access to information on publicly and privately supported clinical studies on a wide range of diseases and conditions. https://clinicaltrials.gov/ct2/results?term = iron+oxide+nanoparticles&Search = Search, accessed: October 2015

[34] U.S. Food and Drug Administration website sharing information about the drug safety and availability. http://www.fda.gov/drugs/drugsafety/ucm440138.htm, accessed: October 2015.

[35] E. A. Neuwelt , B. E. Hamilton , C. G. Varallyay , W. R. Rooney , R. D. Edelman , P. M. Jacobs , S. G. Watnick , Kidney Int. 2009, 75, 465. 18843256 10.1038/ki.2008.496 PMC2643331

[36] M. G. Harisinghani , J. Barentsz , P. F. Hahn , W. M. Deserno , S. Tabatabaei , C. H. van de Kaa , J. de la Rosette , R. Weissleder , N. Engl. J. Med . 2003, 348, 2491. 12815134 10.1056/NEJMoa022749

[37] GRAS Substances (SCOGS) Database from the U.S. Food and Drug Administration website, allowing access to opinions and conclusions from 115 SCOGS reports published between 1972–1980 on the safety of over 370 Generally Recognized As Safe (GRAS) food substances. http://www.fda.gov/Food/IngredientsPackagingLabeling/GRAS/SCOGS/ucm260849.htm, accessed: October 2015.

[38] J. E. Lee , N. Lee , T. Kim , J. Kim , T. Hyeon , Acc. Chem. Res. 2011, 44, 893. 21848274 10.1021/ar2000259

[39] F. Tang , L. Li , D. Chen , Adv. Mater. 2012, 24, 1504. 22378538 10.1002/adma.201104763

[40] D. Shen , J. Yang , X. Li , L. Zhou , R. Zhang , W. Li , L. Chen , R. Wang , F. Zhang , D. Zhao , Nano Lett. 2014, 14, 923. 24467566 10.1021/nl404316v

[41] M. Wu , Q. Meng , Y. Chen , Y. Du , L. Zhang , Y. Li , L. Zhang , J. Shi , Adv. Mater. 2015, 27, 215. 25423915 10.1002/adma.201404256

[42] Y. Chen , Q. Meng , M. Wu , S. Wang , P. Xu , H. Chen , Y. Li , L. Zhang , L. Wang , J. Shi , J. Am. Chem. Soc. 2014, 136, 16326. 25343459 10.1021/ja508721y

[43] F. Chen , H. Hong , Y. Zhang , H. F. Valdovinos , S. Shi , G. S. Kwon , C. P. Theuer , T. E. Barnhart , W. Cai , ACS Nano 2013, 7, 9027. 24083623 10.1021/nn403617j PMC3834886

[44] J. Liu , C. Detrembleur , S. Mornet , C. Jerome , E. Duguet , J. Mater. Chem. B 2015, 3, 6117. 10.1039/c5tb00664c 32262732

[45] M. A. Kostiainen , O. Kasyutich , J. J. Cornelissen , R. J. Nolte , Nat. Chem. 2010, 2, 394. 20414241 10.1038/nchem.592

[46] M. A. Kostiainen , P. Ceci , M. Fornara , P. Hiekkataipale , O. Kasyutich , R. J. Nolte , J. J. Cornelissen , R. D. Desautels , J. van Lierop , ACS Nano 2011, 5, 6394. 21761851 10.1021/nn201571y

[47] J. Liu , M. Yu , C. Zhou , J. Zheng , Materials Today 2013, 16, 477.

[48] J. Liu , M. Yu , X. Ning , C. Zhou , S. Yang , J. Zheng , Angew. Chem. Int. Ed. 2013, 52, 12572. 10.1002/anie.201304465 PMC4127427 24123783

[49] A. A. Burns , J. Vider , H. Ow , E. Herz , O. Penate‐Medina , M. Baumgart , S. M. Larson , U. Wiesner , M. Bradbury , Nano Lett. 2009, 9, 442. 19099455 10.1021/nl803405h PMC6262890

[50] C. Zhou , G. Hao , P. Thomas , J. Liu , M. Yu , S. Sun , O. K. Oz , X. Sun , J. Zheng , Angew. Chem. Int. Ed. 2012, 51, 10118. 10.1002/anie.201203031 22961978

[51] A. Ruggiero , C. H. Villa , E. Bander , D. A. Rey , M. Bergkvist , C. A. Batt , K. Manova‐Todorova , W. M. Deen , D. A. Scheinberg , M. R. McDevitt , Proc. Natl. Acad. Sci. USA 2010, 107, 12369. 20566862 10.1073/pnas.0913667107 PMC2901461

[52] A. M. Smith , H. Duan , A. M. Mohs , S. Nie , Adv. Drug Delivery Rev. 2008, 60, 1226. 10.1016/j.addr.2008.03.015 PMC2649798 18495291

[53] Y. Xing , Q. Chaudry , C. Shen , K. Y. Kong , H. E. Zhau , L. W. Chung , J. A. Petros , R. M. O'Regan , M. V. Yezhelyev , J. W. Simons , M. D. Wang , S. Nie , Nat. Protoc. 2007, 2, 1152. 17546006 10.1038/nprot.2007.107

[54] M.‐K. So , C. Xu , A. M. Loening , S. S. Gambhir , J. Rao , Nat. Biotech. 2006, 24, 339. 10.1038/nbt1188 16501578

[55] M. Yu , J. Zheng , ACS Nano 2015, 9, 6655. 26149184 10.1021/acsnano.5b01320 PMC4955575

[56] J. Liu , M. Yu , C. Zhou , S. Yang , X. Ning , J. Zheng , J. Am. Chem. Soc. 2013, 135, 4978. 23506476 10.1021/ja401612x PMC4127425

[57] S. K. Libutti , G. F. Paciotti , A. A. Byrnes , H. R. Alexander , W. E. Gannon , M. Walker , G. D. Seidel , N. Yuldasheva , L. Tamarkin , Clin. Cancer. Res. 2010, 16, 6139. 20876255 10.1158/1078-0432.CCR-10-0978 PMC3004980

[58] D. Tarn , C. E. Ashley , M. Xue , E. C. Carnes , J. I. Zink , C. J. Brinker , Acc. Chem. Res. 2013, 46, 792. 23387478 10.1021/ar3000986 PMC3686852

[59] R. Bachmann , R. Conrad , B. Kreft , O. Luzar , W. Block , S. Flacke , D. Pauleit , F. Träber , J. Gieseke , K. Saebo , H. Schild , J. Mag. Res. Imag. 2002, 16, 190. 10.1002/jmri.10149 12203767

[60] M. Johannsen , U. Gneveckow , K. Taymoorian , B. Thiesen , N. Waldöfner , R. Scholz , K. Jung , A. Jordan , P. Wust , S. A. Loening , Int. J. Hyperthermia 2007, 23, 315. 17523023 10.1080/02656730601175479

[61] W. C. W. Chan , S. Nie , Science 1998, 281, 2016. 9748158 10.1126/science.281.5385.2016

